# A multimodal deep learning architecture for predicting interstitial glucose for effective type 2 diabetes management

**DOI:** 10.1038/s41598-025-07272-3

**Published:** 2025-07-29

**Authors:** Muhammad Salman Haleem, Daphne Katsarou, Eleni I. Georga, George E. Dafoulas, Alexandra Bargiota, Laura Lopez-Perez, Miguel Rujas, Giuseppe Fico, Leandro Pecchia, Dimitrios Fotiadis, Gatekeeper Consortium, Claudio Caimi, Christian Tamporale, Mirko Manea, Chiara Bonferini, Eugenio Gaeta, Gloria Cea Sánchez, Ioanna Drympeta, Konstantinos Votis, Frans Folkvord, Jordi de Battle

**Affiliations:** 1 School of Engineering, University of Warwick, Coventry, CV4 7AL UK; 2 School of Electronic Engineering and Computer Science, Queen Mary University of London, London, E1 4NS UK; 3 Dept. of Materials Science and Engineering, University of Ioannina, Ioannina, Greece; 4 Faculty of Medicine, University of Thessaly, Volos, Greece; 5 Department of Endocrinology and Metabolic Diseases, University Hospital of Larisa, Larissa, Greece; 6 Universidad Politécnica de Madrid-Life Supporting Technologies Research Group, ETSIT, Madrid, Spain; 7 Università Campus Bio-Medico, Via Álvaro del Portillo, 21, 00128 Roma, Italy; 8 Hewlett-Packard Italiana, Milan, Italy; 9 Information Technologies Institute, Centre for Research and Technology Hellas, Thessaloniki, Greece; 10 PredictBy Research and Consulting, Barcelona, Spain; 11 Tilburg School of Humanities and Digital Sciences, Tilburg, The Netherlands; 12 Hospital Universitari Arnau de Vilanova and Santa Maria, Lleida, Spain; 13 Centro de Investigación Biomédica en Red de Enfermedades Respiratorias (CIBERES), Madrid, Spain

**Keywords:** Multimodal AI, Deep learning, Interstitial glucose prediction, Time series modelling, Computational models, Computational science, Type 2 diabetes

## Abstract

The accurate prediction of blood glucose is critical for the effective management of diabetes. Modern continuous glucose monitoring (CGM) technology enables real-time acquisition of interstitial glucose concentrations, which can be calibrated against blood glucose measurements. However, a key challenge in the effective management of type 2 diabetes lies in forecasting critical events driven by glucose variability. While recent advances in deep learning enable modeling of temporal patterns in glucose fluctuations, most of the existing methods rely on unimodal inputs and fail to account for individual physiological differences that influence interstitial glucose dynamics. These limitations highlight the need for multimodal approaches that integrate additional personalized physiological information. One of the primary reasons for multimodal approaches not being widely studied in this field is the bottleneck associated with the availability of subjects’ health records. In this paper, we propose a multimodal approach trained on sequences of CGM values and enriched with physiological context derived from health records of 40 individuals with type 2 diabetes. The CGM time series were processed using a stacked Convolutional Neural Network (CNN) and a Bidirectional Long Short-Term Memory (BiLSTM) network followed by an attention mechanism. The BiLSTM learned long-term temporal dependencies, while the CNN captured local sequential features. Physiological heterogeneity was incorporated through a separate pipeline of neural networks that processed baseline health records and was later fused with the CGM modeling stream. To validate our model, we utilized CGM values of 30 min sampled with a moving window of 5 min to predict the CGM values with a prediction horizon of (a) 15 min, (b) 30 min, and (c) 60 min. We achieved the multimodal architecture prediction results with Mean Absolute Point Error (MAPE) between 14 and 24 mg/dL, 19–22 mg/dL, 25–26 mg/dL in case of Menarini sensor and 6–11 mg/dL, 9–14 mg/dL, 12–18 mg/dL in case of Abbot sensor for 15, 30 and 60 min prediction horizon respectively. The results suggested that the proposed multimodal model achieved higher prediction accuracy compared to unimodal approaches; with upto 96.7% prediction accuracy; supporting its potential as a generalizable solution for interstitial glucose prediction and personalized management in the type 2 diabetes population.

## Introduction

Type 2 Diabetes Mellitus (T2DM) is characterized by insulin resistance, leading to elevated blood glucose levels, and it accounts for approximately 90% of all diagnosed cases of diabetes^1^. Individuals with T2DM are at 15% higher risk of mortality^2^ as the International Diabetes Federation estimated 537 million people were affected, causing 6.7 million deaths in 2021^3^ and projected to rise up to 783 million by 2045. This not only impacts population health but also imposes a heavy financial strain on both individuals and the global healthcare system, as the American Diabetes Association reported that the total cost of diagnosed diabetes in the United States was 412.9 billion USD in 2022^4^, with a 35% increase in medical costs over the past decade^5^. Efforts to mitigate these costs focus on early detection, effective management strategies, and preventive measures to reduce the incidence and severity of diabetes complications^5^.

Multiple studies have shown that self-monitoring of blood glucose is effective in supporting diabetes management^6^. With the advent of modern wearables and technologies, one study identified high compliance with regular monitoring of blood glucose and other T2DM variables (e.g. diet, physical activity) among individuals using smartphones compared to those using paper diaries^7^. The regular self-monitoring of blood glucose can promote adherence to clinical guidelines for diet and physical activity, resulting in improvements in hemoglobin A1c (HbA1c) levels^8^. Traditionally, regular blood glucose monitoring requires a finger prick test, which is invasive and cumbersome^9^. In contrast, Continuous Glucose Monitoring (CGM) measures the concentration of glucose in the interstitial fluid at regular intervals^10^. While CGM is well established in type 1 diabetes care, its use in T2D is expanding. The ADA Standards of Care in Diabetes 2025 recommend CGM for adults with T2D on glucose-lowering therapies, reflecting its growing role in managing glycemic variability^11^. CGM use in T2D has been associated with reduced risk of severe hypoglycemia, diabetic ketoacidosis, and hospitalizations. Developing accurate glucose predictive models for this population is therefore timely and clinically relevant.

Regular acquisition of blood glucose data holds significant potential for predicting future glucose levels and improving glycaemic control^12^. It also enables the estimation of critical glycaemic events, such as hypoglycaemia (defined as blood glucose levels below 70 mg/dL) and hyperglycaemia (above 180 mg/dL)^13^. However, there are certain challenges associated with predicting blood glucose via CGM values only. Firstly, there is a proven 10-min sensor delay between interstitial fluid glucose and actual blood glucose as measured by CGM^14^. Secondly, CGM systems are susceptible to occasional sensor failure or signal loss, and therefore require reliable strategies to ensure continuity of glucose monitoring during these periods^15,16^.

Advances in artificial intelligence (AI), including traditional machine learning and deep learning techniques, have enabled the development of models that predict interstitial glucose levels 15 to 60 min in advance based on historical CGM-derived glucose readings^17,18^. However, clinical studies suggest the patient-specific differences in glycaemic variability are possible due to underlying conditions (e.g., demographics, comorbidities, or diet plans)^19^ and currently, predictive models do not inform CGM variations based on these underlying conditions.

**Fig. 1:**
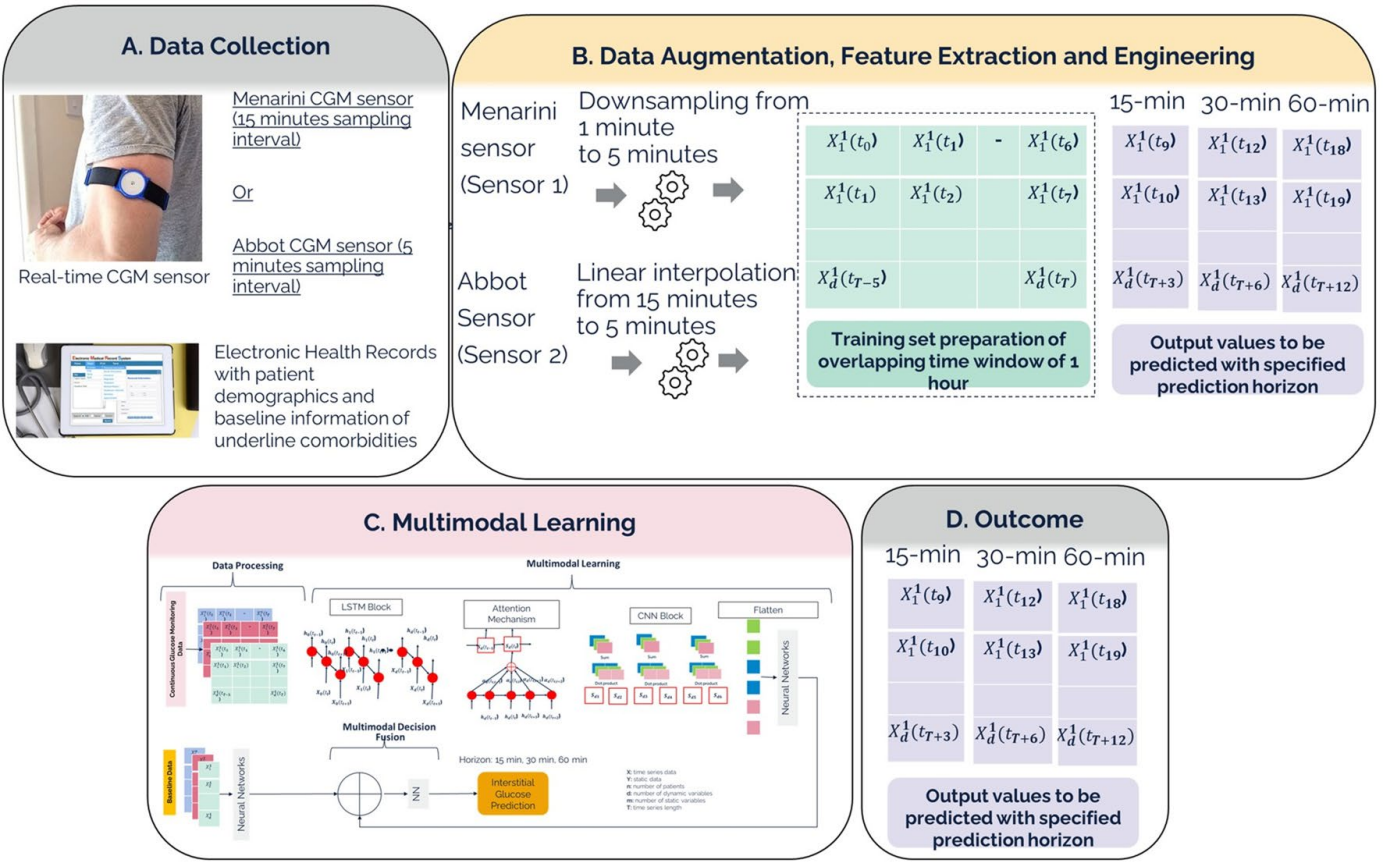
Overall architecture of predicting the continuous interstitial glucose via multimodal architecture.

To address the aforementioned challenge, in this study, glucose levels were monitored using a continuous glucose monitoring (CGM) device, which measures glucose concentration in the interstitial fluid via the subcutaneous tissue. We investigated a multimodal deep learning approach to estimate short-term interstitial glucose levels in individuals with T2D in real-life conditions by informing continuous glucose monitoring (CGM) data with baseline health conditions. The multimodal learning approach involves a sequential deep learning pipeline trained on CGM sequences and context information, while baseline health data serve as auxiliary knowledge to inform CGM variations, which are then combined via a multimodal fusion function. The overall workflow of our architecture has been presented in Fig. 1. The details are presented in the upcoming sections according to the TRIPOD statement as tabulated in Supplementary Table 1.

## Results

### Participants

Table 1 presents the set of input variables along with the characteristics examined in the present predictive modelling study for the 40 subjects. In particular, 15 out of 40 patients used the GlucoMen Day CGM Menarini® sensor (we call Sensor 1) for a monitoring period of 10–19 days, and the remaining 25 patients used the Libre Abbott® system (we call Sensor 2) for a monitoring period of 8–28 days. This has served as initial step for Ambulatory Glucose Profile (AGP) analysis.

**Table 1:** Descriptive characteristics of Central Greece pilot study.

	Feature	Mean ± std
Demographics	Gender (%)	Male: 45 (18)
		Female: 55 (22)
	Age (years)	67 ± 9
Anthropometrics	Weight (kg)	80.42 ± 28.46
	Height (m)	1.63 ± 0.12
	Waist circumference (cm)	104.13 ± 16.5
Biochemical tests	Baseline Blood Glucose (mg/dL)	136.6 ± 45.19
	Baseline HbA1c (%)	7.42 ± 1.11
	Creatinine (mg/dL)	1.99 ± 1.52
	Urea Level (mg/dL)	49.44 ± 30.65
	Total cholesterol (mg/dL)	144.82 ± 33.78
	LDL cholesterol (mg/dL)	67.17 ± 32.11
	HDL cholesterol (mg/dL)	46.86 ± 10.83
	Triglycerides (mg/dL)	203.71 ± 247.43
	White blood cell count (10^3/μL)	7.29 ± 1.83
	Red blood cell count (10^3/μL)	25.26 ± 37.27
	Haematocrit (%)	39.76 ± 8.86
	Plt (× 1000/μL)	206.31 ± 80.53
	SGOT (IU/L)	35.42 ± 32.22
	SGPT (IU/L)	25.99 ± 14.12
	K (mmol/L)	4.57 ± 0.48
	Na (mmol/L)	125.52 ± 42.24

Plt, Platelet Count; SGOT, Serum Glutamic-Oxaloacetic Transaminase; SGPT, Serum Glutamic-Pyruvic Transaminase; HbA1c, Haemoglobin A1c.

**Fig. 2:**
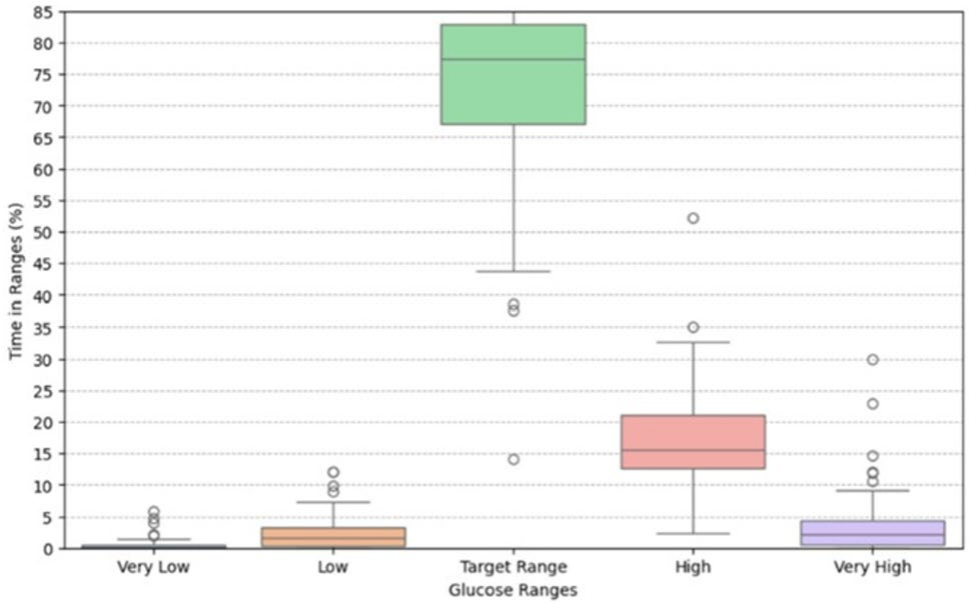
The time in ranges based on AGP report.

Based on AGP analysis, the time spent in clinically defined glucose ranges varied across participants. On average, participants spent approximately 2.68% of the time in low range (glucose < 70 mg/dL) and 0.64% in very low range (glucose < 54 mg/dL), as shown in Fig. 2. Time spent in the high range (glucose > 180 mg/dL) was approximately 20%, and time spent in the very high range (glucose > 250 mg/dL) was approximately 5%. The mean interstitial glucose across the cohort was 146.1 ± 22.98 mg/dL (see Table 2). The results were extracted prior to data curation and preprocessing. Also, the Augmented Dickey-Fuller (ADF) confirmed that the CGM time series for each patient was stationary over the observation period.

**Table 2:** The glucose statistics based on AGP report across all patients using CGM data over the entire period of the study (Data are presented as mean ± standard deviation).

AGP report variables	Mean ± standard deviation
Average glucose (mgdL^-1^)	146.1 ± 22.98%
Glucose management indicator (%)	6.8 ± 0.55%
Glucose variability (%)	31.85 ± 7.36%

### CGM prediction

Table 3 reports the performance of the unimodal prediction pipeline for a 15-min prediction horizon, evaluated using Mean Absolute Percentage Error (MAPE)^20^. The results indicate that emphasizing local CGM features, via weighting mechanisms informed by high CGM contextual variability, enhances prediction accuracy. The Convolutional Neural Network (CNN) driven Long Short Term Memory (LSTM) i.e. CNN-LSTM model with attention achieved the lowest MAPE across both sensors, demonstrating the benefit of incorporating temporal dynamics and adaptive focus on high-variability regions in glucose trends. The statistical significance among difference in MAPE among different architectures of CGM pipeline has been performed via *T test*^21^ and has been presented in Table 4. The results show significant improvement towards adding LSTM and attention mechanism. However, our experiments also suggest that adding more complex layers (e.g. adding multilayer convolutional layers) will add further complexity in the architecture; resulting in decreased performance of the model.

**Table 3:** CGM population model comparison for predicting interstitial glucose with 15-min prediction horizon.

	Menarini sensor MAPE	Abbott sensor MAPE
CNN	14.61 ± 18.98	7.22 ± 8.89
LSTM	14.62 ± 19.91	6.89 ± 9.15
CNN + LSTM	14.51 ± 20.06	7.04 ± 9.25
CNN + LSTM + Attention	14.24 ± 19.42	6.80 ± 9.31

**Table 4:** The statistical significance of difference among different CGM pipelines mentioned in Table 3.

	CNN	LSTM	CNN + LSTM	CNN + LSTM + Attention
Menarini sensor (Sensor 1) statistical significance of difference				
CNN				*
LSTM			*	*
CNN + LSTM		*		*
CNN + LSTM + Attention	*	*	*	
Abbot sensor (Sensor 2) statistical significance of difference				
CNN		***	***	***
LSTM	***		***	***
CNN + LSTM	***	***		***
CNN + LSTM + Attention	***	***	***	

*p_value < 0.05; **p_value < 0.01; ***p_value < 0.001.

### Comparison between multimodal and unimodal architectures

After the CGM pipeline development, we then developed the multimodal architecture by performing additive concatenation between the CGM pipeline and baseline pipeline trained via fully connected dense architecture. The reason for opting for simpler architecture is because the performance of the complex multimodal architecture was constrained by the availability of baseline variables. Including more baseline variables reduced the number of subjects available for training, which in turn impacted model performance. Through our experiments, we have achieved 7 sets of baseline variables which are present with respective number of patient numbers acquired through both Menarini and Abbot sensors. The results have been presented in Table 5.

**Table 5:** List and number of baseline and demographic variables present for both Menarini and Abbot subjects.

Set	Variables	Menarini subjects (Sensor 1)	Abbot subjects (Sensor 2)
0	[‘Age’, ‘Gender’]	15	25
1	[‘Age’, ‘Gender’, 'HbA1c (%)']	8	17
2	[‘Age’, ‘Gender’, 'HbA1c (%)', 'Weight (kg)', 'Height (m)']	6	13
3	[‘Age’, ‘Gender’, 'HbA1c (%)', 'Weight (kg)', 'Height (m)', 'HDL cholesterol (mg/dL)', 'Total cholesterol (mg/dL)']	5	9
4	[‘Age’, ‘Gender’, 'HbA1c (%)', 'Weight (kg)', 'Height (m)', 'HDL cholesterol (mg/dL)', 'Total cholesterol (mg/dL)', 'Blood Glucose (mg/dL)', 'Urea level (mg/dL)']	5	7
5	[‘Age’, ‘Gender’, 'HbA1c (%)', 'Weight (kg)', 'Height (m)', 'HDL cholesterol (mg/dL)', 'Total cholesterol (mg/dL)', 'Blood Glucose (mg/dL)', 'Urea level (mg/dL)', 'K (mmol/L)', 'Haematocrit (%)', 'LDL cholesterol (mg/dL)']	3	5
6	[‘Age’, ‘Gender’, 'HbA1c (%)', 'Weight (kg)', 'Height (m)', 'HDL cholesterol (mg/dL)', 'Total cholesterol (mg/dL)', 'Blood Glucose (mg/dL)', 'Urea level (mg/dL)', 'K (mmol/L)', 'Haematocrit (%)', 'LDL cholesterol (mg/dL)', 'SGOT (IU/L)', 'White blood cell count (10^3/μL)']	2	4

**Fig. 3:**
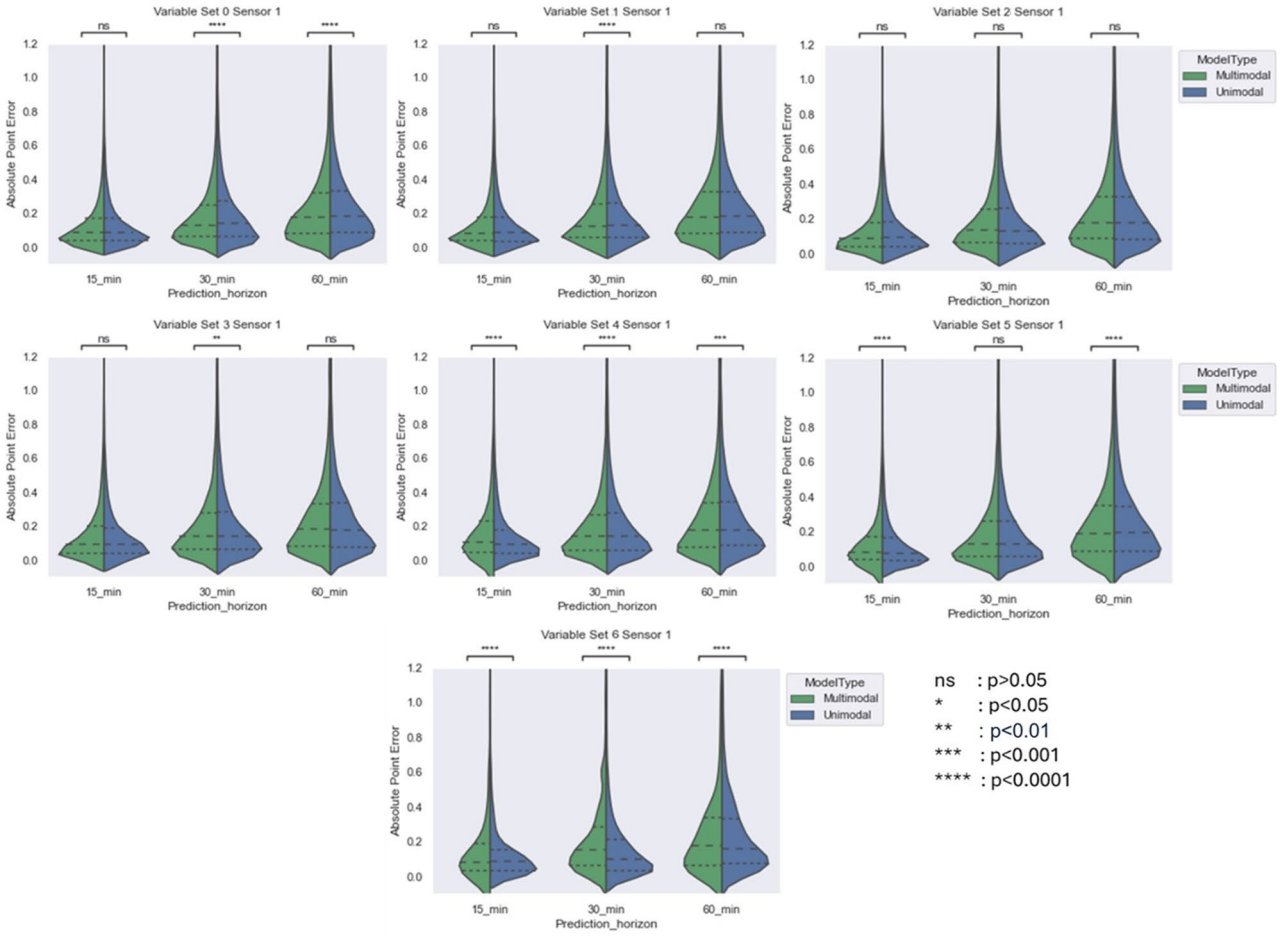
Comparing violin plot of absolute point error for multimodal and unimodal architectures developed for Menarini sensor across different variable sets at three prediction horizon. The violin plot shows the distribution of absolute point error 25%, 50% and 75% quartile via dashed line.

**Fig. 4:**
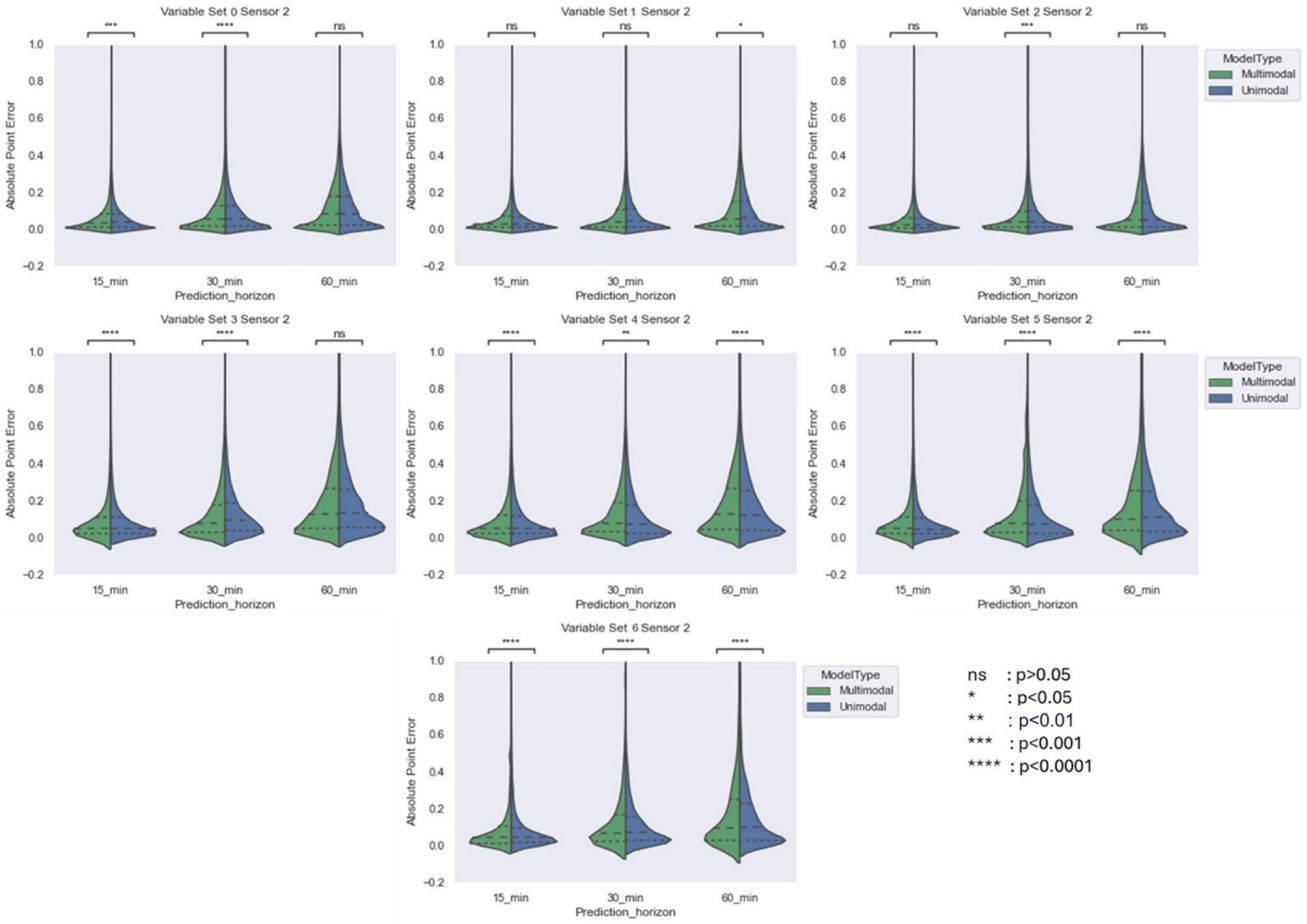
Comparing violin plot of absolute point error for multimodal and unimodal architectures developed for Abbot sensor across different variable sets at three prediction horizon. The violin plot shows the distribution of absolute point error 25%, 50% and 75% quartile via dashed line.

The comparison between unimodal and multimodal architectures has been presented in terms of MAPE as shown in Figs. 3 and 4. We have also tabulated these results not only in terms of overall MAPE, but also in terms of Hyperglycaemic MAPE (where acquired interstitial glucose was greater than 180 mg/dL) as well as Hypoglycaemic MAPE (where acquired interstitial glucose was less than 70 mg/dL). The results have been presented in Table 6. Due to low number of Hypoglycaemic events in Type 2 diabetic subjects acquired from Abbot sensors, the Hypoglycaemic MAPE for these subjects have not been calculated. Besides, due to smaller number of participants for set 5 and onwards, their MAPE results were not stable and therefore not included in the results.

**Table 6:** Comparison of Multimodal architecture performance with Unimodal architecture in terms of Mean Absolute Point Error (MAPE), Hyperglycaemic (interstitial glucose > 180 mg/dL) and Hypoglycaemic (interstitial glucose < 70 mg/dL).

		Sensor 1								
		15 min			30 min			60 min		
	Set	MS 1	UM 1	*p*_value	MS 1	UM 1	*p*_value	MS 1	UM 1	*p*_value
Overall MAPE	0	14.1	14.2		19.6	21.5	***	25.6	26.5	***
	1	14.8	15.1	*	19.9	20.9	***	25.6	25.5	
	2	14.9	15.2	*	20.3	20.6		26.0	26.2	
	3	16.5	16.4		21.9	22.8	***	26.3	26.8	
	4	24.3	15.3	***	21.4	22.8	***	26.2	27.4	**
Hyperglycaemia MAPE	0	12.5	12.5		15.8	17.6	***	21.8	21.3	
	1	11.3	10.4	***	16.0	16.5	*	23.1	24.6	***
	2	11.5	11.7		15.7	17.4	***	21.7	21.6	
	3	11.2	11.1		14.2	15.4	***	21.2	20.9	
	4	13.2	11.7		14.7	16.0	***	20.9	21.3	*
Hypoglycaemia MAPE	0	45.9	45.6		63.8	81.6	***	99.9	105.6	***
	1	52.1	53.3	*	74.7	82.3	***	102.2	97.5	**
	2	47.2	48.8	*	65.5	71.8	***	96.9	101.3	*
	3	55.4	50.3	***	70.8	82.6	***	93.3	95.7	
	4	76.3	42.7	***	69.4	81.6	***	93.8	101.7	***

	Sensor 2									
	Set	MS 2	UM 2	*p*_value	MS 2	UM 2	*p*_value	MS 2	UM 2	*p*_value
Overall MAPE	0	6.7	6.8	***	9.2	9.4	***	12.0	12.0	
	1	5.9	5.8		7.9	8.0	*	10.2	10.4	*
	2	5.3	5.4	*	7.5	7.7	***	9.7	9.7	
	3	11.7	9.5	***	13.6	14.5	***	18.7	18.9	
	4	10.7	9.8	***	13.8	13.3	***	18.0	18.9	***
Hyperglycaemia MAPE	0	10.8	11.8	***	18.6	16.1	***	23.5	23.8	
	1	10.2	10.5		16.3	18.8	***	24.5	24.5	
	2	12.6	14.0	***	17.1	19.4	***	26.6	27.7	***
	3	12.6	11.5	***	16.6	18.1	***	23.6	25.9	***
	4	13.3	12.2	***	18.3	19.0	*	25.6	25.3	

MAPE between acquired interstitial glucose and predicted interstitial glucose. We have include first 5 variable sets from Table 5. Due to low number of Hypoglycaemic events from patients acquired by Abbot sensor, the performance in terms of Hypoglycaemic MAPE has not been included for these patients.

MS 1 = Multimodal Sensor 1; UM 1 = Unimodal Sensor 1;

MS 2 = Multimodal Sensor 2; UM 2 = Unimodal Sensor 2.

Sensor 1: Menarini Sensor; Sensor 2: Abbot Sensor.

*p_value < 0.05; **p_value < 0.01; ***p_value < 0.001.

The results reveals that increasing the prediction horizon negatively impacted cross-validation accuracy. As expected, MAPE increased with longer prediction horizons, with wider APE distributions observed at the 60-min horizon. Despite of constrained availability of baseline variables, the multimodal architecture significantly outperformed the unimodal model for the first four baseline variable sets at both 30-min and 60-min horizons. For the 15-min horizon, the difference in cross-validated MAPE between architectures was not statistically significant for the Menarini sensor (Sensor 1) with baseline sets 3 and 4 due to a smaller sample size (see Table 5), but was significant for the Abbott sensor (Sensor 2), where more subjects were available. In summary, while the unimodal and multimodal models performed comparably at the 15-min horizon, the multimodal architecture showed significantly better performance at 30 and 60 min, likely due to the incorporation of baseline variables that helped inform CGM trends over longer horizons.

### Clinical explainability of prediction performance

**Fig. 5:**
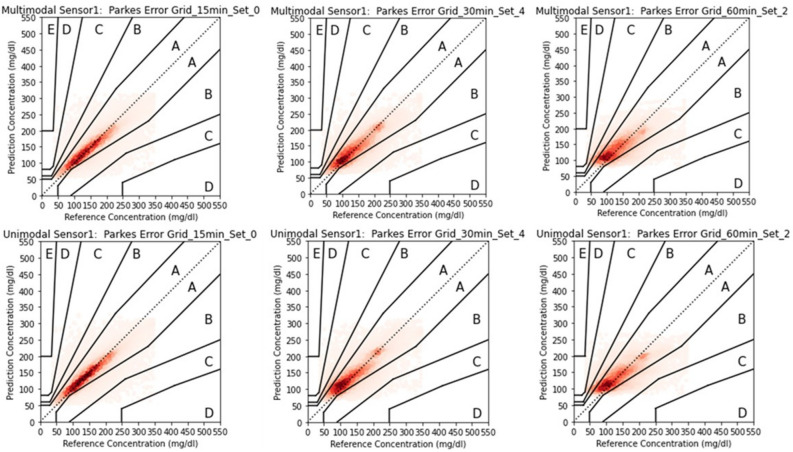
Parkes’ Grid error comparison between multimodal and unimodal architectures at selected variable set for prediction horizon of 15 min, 30 min and 60 min for Menarini sensor.

**Fig. 6:**
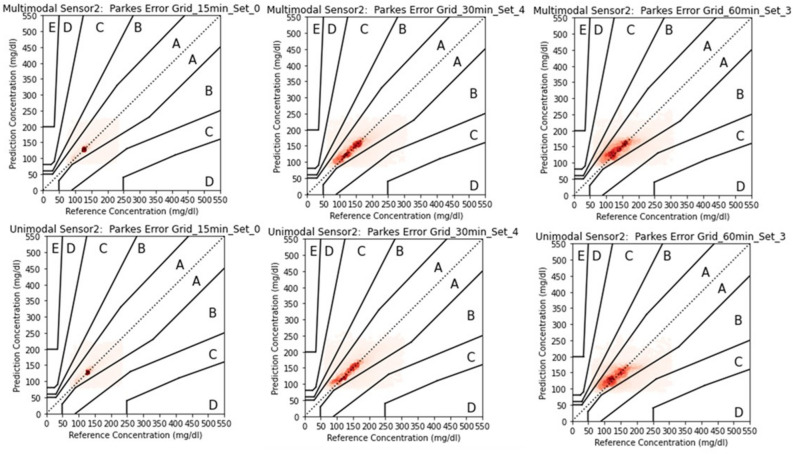
Parkes Grid error comparison between multimodal and unimodal architectures at selected variable set for prediction horizon of 15 min, 30 min and 60 min for Abbot sensor.

We further evaluated the prediction performance of the multimodal architecture in a clinical context using Parkes Error Grid analysis^22^. The Parkes Grid Error was developed to present performance zones for blood/interstitial glucose prediction performance for type 2 diabetic subjects. It has 5 zones ranging from zone A – E with zone A defines “clinically accurate measurements with no impact on clinical actions” and zone B as “altered clinical action, little or no effect on clinical outcome”. The results are tabulated in Table 7 as well as shown in Figs. 5 and 6 for some baseline variable sets. For each prediction horizon visualization, we selected the variable set where the multimodal architecture significantly outperformed the unimodal model (as shown in Figs. 3,4). The results demonstrate that, in all significant cases, multimodal predictions had a higher concentration of values within Zone A of Parkes’ Error Grid for earlier baseline variable sets, across all horizons and for both sensors, indicating greater clinical accuracy. The variable sets with low number of patients do present better performance of unimodal architecture. Moreover, the multimodal models demonstrated improved performance in clinically critical ranges, accurately predicting glucose values as low as 70 mg/dL (hypoglycemia) and as high as 180 mg/dL (hyperglycemia). These findings suggest that incorporating personalized baseline information not only enhances statistical performance, but also improves the clinical reliability of CGM prediction models.

**Table 7:** Percentage distribution comparison of Parkes’ grid error zones for multimodal and unimodal architectures for both Menarini (Sensor 1) and Abbot (Sensor 2).

	15 min					30 min					60 min				
Set	A	B	C	D	E	A	B	C	D	E	A	B	C	D	E
Multimodal Sensor 1															
0	**90.2%**	8.6%	1.1%	0.1%	0.0%	**84.2%**	13.4%	2.2%	0.2%	0.0%	**76.8%**	19.4%	3.6%	0.2%	0.0%
1	**89.9%**	8.8%	1.2%	0.1%	0.0%	**83.6%**	14.0%	2.2%	0.2%	0.0%	**75.8%**	20.6%	3.5%	0.2%	0.0%
2	88.4%	10.2%	1.3%	0.1%	0.0%	**82.5%**	15.1%	2.3%	0.2%	0.0%	**76.1%**	19.6%	4.0%	0.3%	0.0%
3	**88.4%**	9.9%	1.6%	0.2%	0.0%	**81.5%**	15.4%	2.9%	0.3%	0.0%	74.0%	21.2%	4.6%	0.2%	0.0%
4	84.5%	10.4%	4.4%	0.7%	0.0%	**81.0%**	15.7%	3.0%	0.3%	0.0%	73.6%	21.6%	4.6%	0.2%	0.0%
Unimodal Sensor 1															
0	89.7%	9.1%	1.0%	0.1%	0.0%	82.6%	15.4%	1.8%	0.1%	0.0%	76.8%	19.2%	3.7%	0.3%	0.0%
1	89.7%	8.9%	1.3%	0.1%	0.0%	83.5%	14.2%	2.1%	0.2%	0.0%	74.1%	22.2%	3.5%	0.2%	0.0%
2	**89.4%**	9.1%	1.3%	0.1%	0.0%	81.8%	15.9%	2.2%	0.1%	0.0%	75.1%	20.6%	4.2%	0.1%	0.0%
3	87.4%	10.8%	1.6%	0.2%	0.0%	80.7%	16.6%	2.4%	0.2%	0.0%	**74.3%**	20.8%	4.6%	0.3%	0.0%
4	**86.6%**	11.8%	1.4%	0.2%	0.0%	80.7%	16.7%	2.5%	0.2%	0.0%	**73.7%**	21.3%	4.8%	0.2%	0.0%
Multimodal Sensor 2															
0	**96.7%**	3.3%	0.0%	0.0%	0.0%	**95.2%**	4.8%	0.1%	0.0%	0.0%	**93.0%**	7.0%	0.0%	0.0%	0.0%
1	**97.5%**	2.5%	0.0%	0.0%	0.0%	**96.0%**	4.0%	0.1%	0.0%	0.0%	**93.5%**	6.5%	0.1%	0.0%	0.0%
2	**97.6%**	2.4%	0.0%	0.0%	0.0%	**96.4%**	3.6%	0.1%	0.0%	0.0%	**94.0%**	6.0%	0.1%	0.0%	0.0%
3	92.3%	6.2%	1.5%	0.0%	0.0%	**91.2%**	7.9%	0.9%	0.0%	0.0%	**85.4%**	13.4%	1.2%	0.0%	0.0%
4	93.2%	5.6%	1.2%	0.0%	0.0%	90.1%	8.9%	1.0%	0.0%	0.0%	83.6%	14.2%	2.2%	0.0%	0.0%
Unimodal Sensor 2															
0	96.6%	3.3%	0.0%	0.0%	0.0%	94.7%	5.2%	0.0%	0.0%	0.0%	92.7%	7.2%	0.0%	0.0%	0.0%
1	97.5%	2.4%	0.0%	0.0%	0.0%	96.0%	4.0%	0.1%	0.0%	0.0%	93.5%	6.4%	0.1%	0.0%	0.0%
2	97.4%	2.5%	0.0%	0.0%	0.0%	96.0%	4.0%	0.0%	0.0%	0.0%	93.9%	6.1%	0.0%	0.0%	0.0%
3	94.4%	5.0%	0.6%	0.0%	0.0%	89.9%	8.9%	1.2%	0.0%	0.0%	83.6%	14.9%	1.5%	0.0%	0.0%
4	94.3%	5.0%	0.7%	0.0%	0.0%	**90.4%**	8.6%	1.0%	0.0%	0.0%	**84.4%**	14.3%	1.3%	0.0%	0.0%

## Discussion

### Interpretation

In this study, we presented a novel multimodal architecture for predicting interstitial glucose with prediction horizon of 15, 30 and 60 min based on 30-min CGM variation sampled at 5 min along with baseline information of Type 2 subjects representing physiological status. Our novel multimodal architecture addresses several key questions, and, to the best of our knowledge, this is the first study to develop a multimodal architecture for predicting interstitial glucose of Type 2 diabetic subjects with personalized prior information. This study has the potential to estimate the chronic events such as hyperglycaemia and hypoglycaemia in real time based on personalized information with high clinical reliability. The technique was tested on a specific dataset of CGM values collected via two different sensors (Menarini sensor 1 with 1-min sampling downsampled to 5 min and Abbot sensor 2 with 15-min sampling which were upsampled to 5 min for consistency) from an elderly population with Type 2 diabetes whose baseline information representing their physiological status was also provided. The use of this original dataset highlights the robustness of the method in handling the complexities and challenges inherent in cohort interstitial glucose prediction and its accuracy at the personalized level.

Initially, we developed the unimodal architecture in which we developed the training pipeline based solely on CGM values. We first utilized basic deep learning blocks (such as convolutional neural networks (CNN), and long short-term memory (LSTM)) which was followed by adding the attention mechanism in order to train CGM sequential features while highlighting them based on temporal context. This improved the interstitial glucose prediction performance at a prediction horizon of 15 min in terms of Mean Absolute Percentage Error (MAPE) as separate architectures were developed for CGM values acquired from two different sensors; thus compared separately.

The CGM training pipeline was then concatenated with the personalized baseline information pipeline to inform variations in the CGM training through additive concatenation, leading towards multimodal information. The multimodal architecture performance was compared with the unimodal architecture which involved a CGM pipeline only for predicting with prediction horizon of 15, 30 and 60 min. The leave-day cross validation protocol was introduced in which a day was kept out for testing purposes whereas the rest of days were used as training set with day-window sliding after every cycle. The prediction performance was also compared in terms of clinical significance using *Parkes’ Grid error*, a graphical tool used to evaluate both accuracy and clinical relevance of glucose predictions in Type 2 diabetes subjects. The results show that informing the CGM variations based on personalized baseline information improves the prediction performance at the cohort level.

The multimodal model architecture significantly outperformed the unimodal architecture in terms of MAPE for first four baseline variable sets. For a prediction horizon of (i) 15 min, (ii) 30 min and (iii) 60 min, the MAPE was between (i) 14–16 mg/dL, (ii) 19–21 mg/dL and (iii) 25–26 mg/dL respectively compared to unimodal architectures with MAPE between (i) 14–16 mg/dL, (ii) 21–23 mg/dL and (iii) 26–27 mg/dL respectively. Besides, there has been higher concentration in zone A of Parkes’ Grid error for multimodal architecture of these variable sets which shows high clinical significance. Of course the increase of prediction horizon reduced the prediction performance in terms of both MAPE and Parkes’ Grid error. Nevertheless, the performance drop for multimodal architecture while moving from a prediction horizon of 15 min to 60 min was lower compared to unimodal architectures as shown in APE distribution in Figs. 3 and 4.

In this study, we also observed the MAPE in terms of chronic events such as Hyperglycaemia and Hypoglycaemia. We observed that the performance of predicting high interstitial glucose values (Hyperglycaemia) was even better within 15 min and 30 min prediction horizon. This results in significance of our multimodal architecture in the case of Type 2 diabetes as there had been high number of hyperglycaemic events in Type 2 diabetes. On the other hand, our multimodal architecture performance was relatively poor in predicting blood glucose in hypoglycaemic range. This is because there had been low number of hypoglycaemic events occurred in Type 2 diabetes; resulting in data imbalance problem.

### Limitations

This study had some limitations. Firstly the bottleneck associated with multimodal architecture performance for large baseline variable set was their availability. The variable sets were defined based on their availability with respect to the individual patients. As shown in Table 5, increase in number of baseline variables reduced the number of patients as there were only few patients with every baseline information. The low number of patients actually impacted the multimodal architecture performance as it worked better for a higher number of patients. Secondly, the CGM values collected for individual subjects were not consistent as data acquisition varied from total number from 4 to 10 days. Our upcoming studies may incorporate augmenting the dataset based on probabilistic distribution at the individualized physiology. Thirdly, the dataset size was relatively small (n = 40) and unevenly split across the two CGM devices (Sensor 1 and Sensor 2). Although population models were trained separately for each sensor to account for device-specific variation, domain adaptation techniques were not applied in this study. Future work will explore such approaches to enhance model generalizability across CGM systems and broader T2D populations.

In this study, we opt for additive concatenation approach due to the limitation of size of the dataset. This is because adding model complexity (such as transformer mechanism) resulted in prediction performance. As a part of our future studies, we aim to develop the advanced Graphical Neural Networks which can train the CGM variations based on counterfactual analysis of underline comorbidities. Besides, due to limitations in terms of dataset size, the performance depreciation was observed while increasing the underline comorbidities due to their availability with limited patients.

## Methods

### Study protocol

This study is part of the GATEKEEPER strategy for the Multinational Large-Scale Piloting of an eHealth Platform^23^. The data were collected in the frame of the Central Greece High Complexity Phase I pilot study: a non-interventional, prospective observational study. The applied eligibility criteria encompass elderly patients with T2D and comorbidities, aged 60 years and older. These patients belonged to the intermediate and poor health groups according to clinical guidelines mentioned in^24^. These groups had 3 or more non-diabetic chronic illnesses with mild to severe cognitive impairment. Specifically, people with T2D participated in Phase I, using either the GlucoMen Day Menarini® Continuous Glucose Monitoring (CGM) system (15-min sampling interval) or the Libre Abbot® system (5-min sampling interval) for a monitoring period of up to 4 weeks.

Calibration is the key difference among both sensors. Abbott’s FreeStyle Libre is factory calibrated and remains stable throughout its lifespan, while Menarini’s GlucoMen Day requires periodic user calibration, which can introduce variability. Sensor placement also differs: Abbott sensors are worn on the back of the arm, and Menarini sensors may be placed elsewhere, affecting accuracy due to variations in skin thickness, blood flow, and fat. Additionally, Abbott’s sensor measures every 15 min, while Menarini’s system measures every minute, which can impact data quality, especially during rapid glucose changes. Both sensors can be influenced by environmental factors like temperature, sweating, and physical activity, with Menarini’s sensor being more sensitive to skin perspiration. These factors, along with individual physiological differences, contribute to discrepancies between the two devices.

Patients with severe hearing or vision problems or any other acute or chronic condition that would limit the ability of the user to participate in the study were excluded^25^. All the data collection methods were performed in accordance with the relevant guidelines of the Institutional Review Board of Larisa University Hospital, which are aligned with the Declaration of Helsinki^26^. The names of all participants and other HIPAA identifiers^27^ have been removed prior to data sharing. Furthermore, informed consent has been obtained from all participants and/or their legal guardians. The timeline for the study protocol (incl. ethical approval, study design, data acquisition and integration into GATEKEEPER high performance big data platform) has been presented in Supplementary Fig. 1. For more information about study participants, please check clinical trials.gov ID NCT05461716.

### Data curation and preprocessing

We applied exploratory techniques to visualize each patient’s CGM data, including histograms, autocorrelation plots, partial autocorrelation plots, and the Augmented Dickey-Fuller (ADF) test. We also checked for duplicates and outliers in each time series. To handle missing values in the glucose sensor data, linear interpolation was applied to ensure continuity in the time series. Specifically, missing values in the glucose sensor readings were imputed using linear interpolation. Additionally, for patients using the Abbott sensor, the data was kept at its original sampling frequency of 15 min, as provided by the sensor. However, for patients using the Menarini sensor, the data was resampled to a 5-min interval. Patients were excluded from the analysis if more than 50% of their CGM data was missing, either due to sensor dropouts or user non-compliance. This threshold was set to ensure that the remaining data was sufficiently complete to maintain the integrity and reliability of the analysis. Each type of data was processed separately before merging. Min–Max scaling was applied to normalize the CGM data to a range of [0, 1], while no normalization techniques were applied to the baseline variables.

### Outcome and predictors definition

The output of the predictive model describes the concentration of glucose concentration in the interstitial fluid at time t + *P*H for a prediction horizon (PH) equal to 15, 30 or 60 min. The univariate models’ input comprises the history of interstitial glucose concentration values, as recorded by the CGM system. In the case of multimodal models, the input includes additionally specific EHR variables. T2D participated in this study used either the GlucoMen Day Menarini® Continuous Glucose Monitoring (CGM) system with sampling interval of 15 min; or the Libre Abbot® system with sampling interval of 5 min; for a monitoring period of up to 4 weeks. Besides, for informing interstitial glucose variation with underline comorbidities, we have also included baseline variables representing these comorbidities as predictors.

### Multimodal architecture for CGM prediction

We developed multimodal architectures built upon deep neural networks which have the capability to model real-time CGM variations while informing these variations via appropriate information fusion methods. The CGM variations have been informed by patient electronic health records (e.g. demographics, or anthropometrics, as shown in Table 1). CGM data had been acquired from T2DM patients under real-time conditions. We compiled and compared the results by performing cross validation using the first 30 min of CGM data for training and predicting the CGM values after prediction horizon of (i) 15 min, (ii) 30 min and (iii) 60 min. The training and test sets were derived based on setting the interval length of test set of one day which were sliding from beginning till end of the dataset.

At the first instance, we derived the CGM-only trained population model to find optimal deep neural network architecture; we call it unimodal architecture.

At the second instance, we derived the deep neural networks pipeline which was trained on the T2D patient baseline information. First, we involved two baseline variables which were present in most of the subjects using Abbot and Menarini CGM devices. The inclusion of more baseline variables led to a reduction in the number of available subjects. At the end, there were only 6 subjects who had 14 baseline variables. The list has been presented in Table 5. This enabled us to develop 7 multimodal architectures due to 7 baseline variables subsets. The output of the baseline deep neural network was then fused into the CGM-only training pipeline (unimodal architecture) via additive fusion methods followed by the deep neural network training on CGM variation features informed by the baseline network.

We trained and compared unimodal and multimodal architectures across 7 variable sets for predicting CGM values for the aforementioned prediction horizons of 15, 30 and 60 min. The comparison for both sensors was performed separately using violin plot, showing the distribution of absolute point errors between predicted CGM values and real CGM values, along with quartile markings at 25%, 50% and 75%. Due to instability in the model performance for baseline variable set 5 and onwards, we included the performance comparison from variable set 0 to variable set 4 only.

### Clinical explainability of prediction performance

We further assessed the multimodal architecture prediction performance under clinical settings based on *Parkes grid error*^22^. The Parkes ‘grid error classifies the scatter plot of predicted interstitial glucose and reference interstitial glucose for type 2 diabetic subjects in five different zones: A, B, C, D and E. The estimation in zone A would be considered as ideal, whereas estimation in zone B would be considered as clinically acceptable.

### Model development

**Fig. 7:**
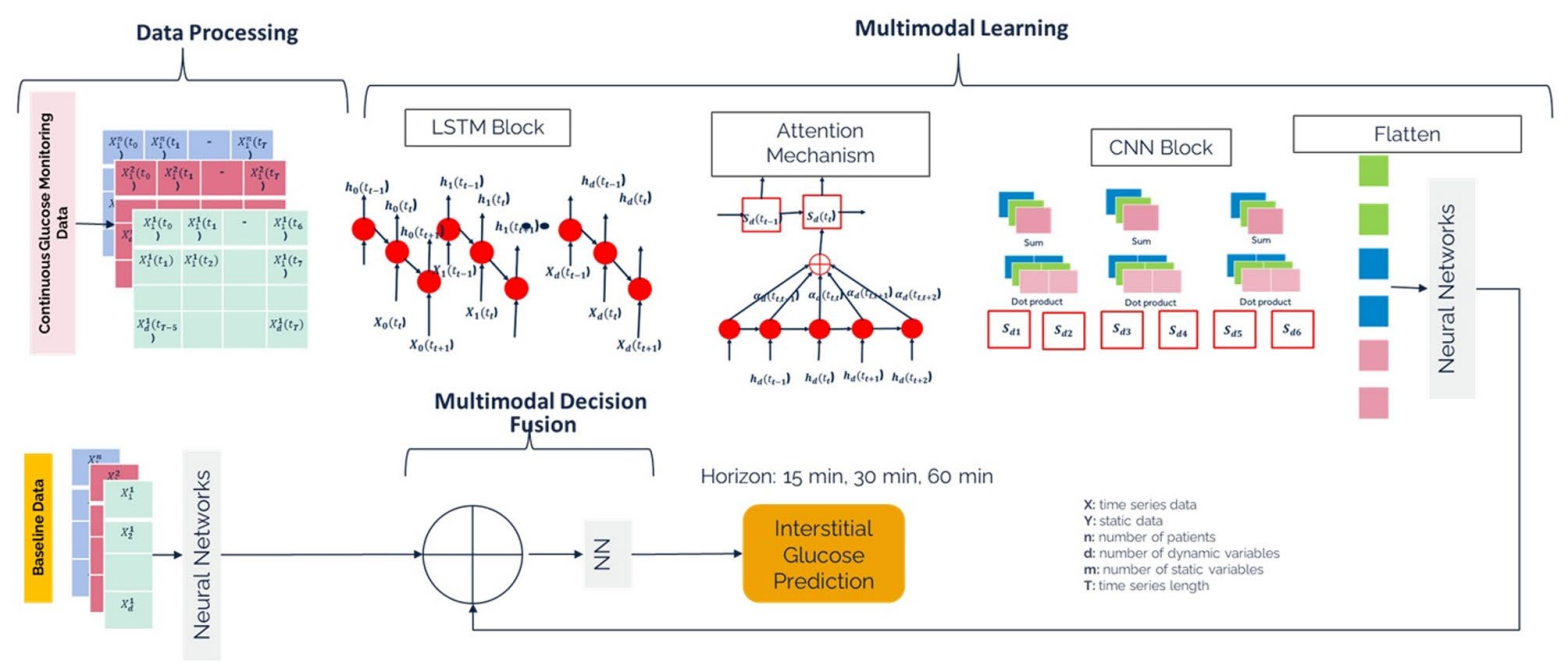
Multimodal Architecture for predicting blood glucose.

The block diagram of architecture has been presented in Fig. 7. The architecture has been designed to predict the interstitial glucose at defined time horizon based on (i) time series historical values from continuous glucose monitoring (CGM) and (ii) static baseline health record information. Let CGM is represented as $X$ has the dimension of $n$ x T; where $n$ is number of users and T is the length of temporal dimension of the CGM input. The CGM values had been acquired from multiple users across different number of days. Each user had been provided one out of two types of CGM devices. One type of device had sampling frequency of 5 min whereas other type of device had sampling frequency of 15 min. For users with a 15-min sampling interval (i.e., Abbot Sensor 2), CGM time series were upsampled to 5 min intervals using linear interpolation. Similarly, Menarini sensor with sampling interval of 1-min was downsampled to 5 min to ensure consistent temporal resolution with Abbot Sensor 2. Considering data acquisition spanning around a couple of days, we performed the data window scheme acquiring ${t}_{0}-{t}_{5}$, ${t}_{1}-{t}_{6}$, ${t}_{{T}^{n}-5}-{t}_{{T}^{n}}$; where ${T}^{n}$ is total number of samples for user $n$. We used 30-min sample to predict interstitial glucose with time horizon of (i) 15 min, (ii) 30 min and (iii) 60 min.

As mentioned in Figs. 1 and 7, the local and temporal features of CGM have been acquired by 1D BiLSTM network with attention layer followed by staked 1D CNN layer. The model initially prepares the CGM values based on the aforementioned window scheme. Concurrently, the baseline data is treated as separate input which is preprocessed and learned separately using a set of dense layers to extract representative deep features. After acquisition of local and temporal features from CGM and representative deep features from baseline data, we added a fusion layer concatenating both types of features followed by a dense layer with sigmoid activation for regressing the CGM values.

To model the temporal context of the CGM data, we first deployed BiLSTM layers; allowing temporal patterns from CGM to be extracted. The core structure of the LSTM cell is the use of three gates i.e. the input gate (${i}_{{t}_{T}}$), the forget gate (${f}_{{t}_{T}}$), and the output gate (${o}_{{t}_{T}}$). These gates control the update, maintenance, and deletion of information contained in a cell state ${C}_{{t}_{T}}$; ${C}_{{t}_{T-1}}$, and ${\widetilde{C}}_{{t}_{T}}$ respectively whereas ${h}_{{t}_{T}}$ is the value of the hidden layer at time ${t}_{T}$. $\theta$ s represent set of weight matrices and $b$ s represent set of biases vectors which are updated following backpropagation algorithm with each temporal iteration. Besides, $\theta$ s and the $b$ s are the set of weight matrices and biases vectors, respectively, updated following the backpropagation through time algorithm. In addition, $\otimes$ represents the Hadamard product; $\sigma$ is the standard logistic sigmoid function; $\oplus$ is the concatenation operator; and $\varphi$ the output activation function. Equations (1)–(7) give the transmission of information in the memory cell at each step.

1 $${f}_{{t}_{T}}=\sigma ({\theta }_{f}\cdot \left[{h}_{{t}_{T-1}}, {X}_{{t}_{T}}\right]+{b}_{f})$$

2 $${i}_{{t}_{T}}=\sigma ({\theta }_{i}\cdot \left[{h}_{{t}_{T-1}}, {X}_{{t}_{T}}\right]+{b}_{i})$$

3 $${\widetilde{C}}_{{t}_{T}}=tanh({\theta }_{c}\cdot \left[{h}_{{t}_{T-1}}, {X}_{{t}_{T}}\right]+{b}_{c})$$

4 $${C}_{{t}_{T}}={f}_{{t}_{T}}\otimes { C}_{{t}_{T-1}}\oplus {i}_{{t}_{T}}\otimes {\widetilde{ C}}_{{t}_{T}}$$

5 $${o}_{{t}_{T}}=\sigma ({\theta }_{o}\cdot \left[{h}_{{t}_{T-1}}, {X}_{{t}_{T}}\right]+{b}_{o})$$

6 $${h}_{{t}_{T}}= {o}_{{t}_{T}}\otimes \boldsymbol{ }tanh ({C}_{{t}_{T}})$$

7 $${y}_{T}= \varphi ({\theta }_{y}{h}_{{t}_{T}}+{b}_{y})$$

In order to take the advantage of temporal context in both directions, we deployed *BiLSTM* which combines input from two separate hidden LSTM layers in opposite direction to the same output. Let’s consider ${X}^{1}({t}_{0:5})$=( ${X}^{1}({t}_{0}$), ${X}^{1}({t}_{1}$), ${X}^{1}({t}_{2}$), ${X}^{1}({t}_{3}$), ${X}^{1}({t}_{4}$), ${X}^{1}({t}_{5}$) ); for which LSTM hidden layer becomes ${\overrightarrow{h}}_{t}^{n}=\left({\overrightarrow{h}}_{{t}_{0}}^{n},{\overrightarrow{h}}_{{t}_{1}}^{n},{\overrightarrow{h}}_{{t}_{2}}^{n},{\overrightarrow{h}}_{{t}_{3}}^{n},{\overrightarrow{h}}_{{t}_{4}}^{n},{\overrightarrow{h}}_{{t}_{5}}^{n}\right)$ towards forward hidden sequence and ${\overleftarrow{h}}_{t}^{1}=\left({\overleftarrow{h}}_{{t}_{0}}^{1},{\overleftarrow{h}}_{{t}_{1}}^{1},{\overleftarrow{h}}_{{t}_{2}}^{1},{\overleftarrow{h}}_{{t}_{3}}^{1},{\overleftarrow{h}}_{{t}_{4}}^{1},{\overleftarrow{h}}_{{t}_{5}}^{1}\right)$ towards backward hidden sequence. Thus, Eq. (7) is now driven as:

8 $${\overrightarrow{h}}_{{t}_{T}}^{n}=\sigma \left({\theta }_{{\overrightarrow{h}}_{T}^{n}} . \left[{\overrightarrow{h}}_{{t}_{T-1}}^{n},{X}^{n}\left({t}_{T}\right)\right]+{b}_{{\overrightarrow{h}}_{T}^{n}}\right)$$

9 $${\overleftarrow{h}}_{{t}_{T}}^{n}=\sigma \left({\theta }_{{\overleftarrow{h}}_{T}^{n}} . \left[{\overleftarrow{h}}_{{t}_{T+1}}^{n},{X}^{n}\left({t}_{T}\right)\right]+{b}_{{\overleftarrow{h}}_{T}^{n}}\right)$$

10 $${(\overrightarrow{h}}_{{t}_{0}}^{n},{\overleftarrow{h}}_{{t}_{0}}^{n})\dots {(\overrightarrow{h}}_{{t}_{T}}^{n},{\overleftarrow{h}}_{{t}_{T}}^{n})=BiLSTM({X}^{n}\left({t}_{0}\right), {X}^{n}\left({t}_{1}\right), ... , {X}^{n}({t}_{5})$$

11 $${y}_{t}^{n}= \varphi ({\theta }_{{y}_{t}^{n}{\overrightarrow{h}}_{T}^{n}}{\overrightarrow{h}}_{{t}_{T}}^{n}+{{\theta }_{{y}_{t}{\overleftarrow{h}}_{T}^{n}}{\overleftarrow{h}}_{{t}_{T}}^{n}+b}_{{y}_{t}^{n}})$$

The output ${y}_{t}^{n}$ is used as an input to the *self-attention layer* which had been deployed to highlight the local CGM features under consideration based on the temporal context. This can be represented by ${\sigma }^{a}{\left({q}^{a},{v}^{a}\right)}_{t,{t}{\prime}}$ which is the softmax function between query (context) of the attention layer ${q}^{a}$ and value of attention layer ${v}^{a}$ at time $t$ and ${t}{\prime}$.

12 $${\sigma }^{a}{\left({q}^{a},{v}^{a}\right)}_{t,{t}{\prime}}=\frac{{e}^{dot({q}_{t}^{a},{v}_{{t}{\prime}}^{a})}}{\sum_{t=0}^{{l}_{f}}{e}^{dot({q}_{t}^{a},{v}_{{t}{\prime}}^{a})}}$$

where ${l}_{f}$=6 is the number of output units of the BiLSTM later. Since it is the self-attention mechanism, the input to both is ${y}_{t}^{n}$.

The 1D *Convolutional Neural Network (CNN)* blocks had been deployed to model the local features provided by self-attention layer based on temporal context of the CGM. 1D CNN can learn attention driven temporal context time series univariate data where convolution is done separately along the time dimension for every input vector. Formally if input ${\sigma }^{a}{\left({q}^{a},{v}^{a}\right)}_{t,{t}{\prime}}\in {\mathbb{R}}^{{l}_{f}\times 1}$ and kernel $K$ is $m\times 1$ then convolutional output in new feature space would be ${\sigma }^{{\prime}a}{\left({q}^{a},{v}^{a}\right)}_{t,{t}{\prime}}\in {\mathbb{R}}^{[\frac{{l}_{f}-m}{d+1},1]}$, where $d$ is the step size. Based on number of filters, the CNN expands the attention output to more abstract and informative features, called feature maps. Each value ${p}_{i}$ of the feature map $p$ is then fed into activation function, $\varnothing$, to calculate ${p}_{i}=\varnothing \left({K}^{T}\times {\sigma }^{a\left(i:i+j-1\right)}+b\right)$, where activation function $\varnothing$ is non-linear activation function $RELU\left(x\right)=\text{max}(o,x)$, $b$ is the bias and ${\sigma }^{a\left(i:i+j-1\right)}$ is the $j$ observation from ${\sigma }^{a}.$ The CNN networks have been followed by 20% dropout to avoid overfitting. The kernel in the convolutional layer had been initialized by Glorot Uniform which initializes the convolutional weights based on uniform distribution within range [-limit, limit] where limit = $\sqrt{\frac{6}{{f}_{in}+{f}_{out}}}$ where ${f}_{in}$ is number of input units and ${f}_{out}$ is number of output units.

As empirical experimentation, we put size of kernel K as 3 for CNN with number of filters as 100. The CNN network was then followed by 10% dropout.

The *multimodal fusion network* allows to fuse the representations learned from the CGM values and the baseline data. Considering that the learned representation from CGM values (i.e. CNN output) is ${Z}^{1}$ and the learned representations from fully connected neural networks trained on baseline data is ${Z}^{2}$. The fusion of both representations are learned by multi-layer fully connected neural network. This can be represented as:

13 $${Z}^{3}=G({Z}^{1}\oplus {Z}^{2},{W}_{3})$$

where $\oplus$ is the fusion operator, ${W}_{3}$ is the matrix of trainable weights and $G$ is the multilayer fully connected neural network. Following the multimodal fusion, we deployed dense layer regressor to predict the interstitial glucose with specified prediction horizon. The regressor is a fully connected neural network followed by sigmoid function. The final results of the regressor and classifier are represented as ${\widehat{Y}}_{{T}^{n}}^{n}\in {T}^{n}\times 1$ where ${T}^{n}$ is total number of time samples for the subject $n$.

The *objective loss function* of estimating interstitial glucose is log likelihood function represented as:

14 $$\mathcal{L}=\sum_{k=1}^{n}\sum_{i=1}^{{T}_{k}}\left({\widehat{Y}}_{i}^{k}-log\left(\sum_{j\epsilon {t}_{i}}exp({\widehat{Y}}_{j}^{k})\right)\right)$$

where ${t}_{i}$ is the prediction horizon for estimating interstitial glucose of the subject $k$. Noting that loss function is the summation of predicting interstitial glucose for every subject $k$ with their respective samples ${T}_{k}$.

### Model evaluation

Mean Absolute Point Error (MAPE) has been selected to evaluate the model which measures average magnitude of error produced by a model with the advantage of scale-independency and interpretability^20^. It can be calculated as:

15 $$MAPE=100\frac{1}{n}\sum_{t=1}^{n}\left|\frac{CG{M}_{t}^{A}-CG{M}_{t}^{P}}{CG{M}_{t}^{A}}\right|$$

where $CG{M}_{t}^{A}$ is actual CGM value and $CG{M}_{t}^{P}$ is the predicted CGM value.

Τo evaluate model performance, we employed a leave-one-day-out cross-validation approach. In this method, each day’s data was sequentially designated as the test set while the remaining data was used for training. This sliding window technique ensured that each data point was tested at least once while maximizing the amount of training data available for each iteration. For each iteration, data preceding the test day and data following the test day were combined to form the training set, while the designated day was held out as the test set. This data partitioning strategy is commonly used in time-series forecasting studies where temporal dependencies are critical.

The multimodal architecture training and validation had been implemented on GATEKEEPER Big Data platform where all the data from the pilot has been hosted and deep learning packages have been trained and tested in the platform. The total training time was 1 min to run 50 iterations in each cross-validation cycle.

## Related work

Deep learning has emerged as a leading approach in interstitial glucose predictions, with a primary focus on applications in Type 1 Diabetes Mellitus (T1DM)^28^. Initial work using LSTM-based models on the OhioT1DM dataset^29,30^ showed limited gains over feature-engineered traditional Machine Learning (ML) methods. More sophisticated architectures, including attention-based Gated Recurrent Units (GRU)s^31^ and CNNs^32^, have since demonstrated improved performance across T1D, T2D, and gestational diabetes datasets.

A growing number of studies aim to improve individual-level prediction accuracy while ensuring generalizability across diverse populations and data sources. In the context of T1D, Zhu et al.^33^ utilised meta-learning and evidential deep learning (i.e., including an attention-based bidirectional Recurrent Neural Networks (RNN) and evidential regression) to quantify uncertainty and personalize glucose forecasting. Daniels et al.^34^ introduced a multitask learning architecture that jointly models shared and individual-specific representations of glucose dynamics in T1D patients. Regarding T2D, Deng et al.^35^ employed deep transfer learning with data augmentation to improve robustness under limited data conditions. Sun et al.^36^ developed a Bayesian structural time series model that incorporates clinical data priors (i.e., anthropometric and biochemical characteristics) to address inter-individual variability in T2D. Similarly, Yang et al.^37^ proposed a clustering-based domain adaptation approach, enabling more personalized modelling by aligning latent representations across patient subgroups.

Complementary to models based solely on CGM, Montaser et al.^38^ proposed a seasonal stochastic local modelling framework that explicitly incorporates variable-length, time-stamped events such as meals and physical activity. This work underscores the relevance of irregular but clinically significant behavioural factors in interstitial glucose prediction. Other contributions have emphasized model interpretability in multivariate glucose predictive modelling; a graph-attentive RNN (GARNN) framework^39^ captures detailed interactions among CGM and self-reported event data, enhancing both prediction accuracy and transparency.

## Conclusion

In this paper, we designed and developed a novel generalized multimodal architecture based on 30-min CGM values informed by baseline physiological information of Type 2 diabetic patients for predicting CGM values with prediction horizon of 15, 30 and 60 min. To the best of our knowledge, this is the first study of predicting interstitial glucose values where CGM variation were informed by individual physiology. Compared to unimodal architecture, we achieved the mean absolute point error of (i) 14–16 mg/dL, (ii) 19–21 mg/dL and (iii) 25–26 mg/dL for predicting CGM values with prediction horizon of 15, 30 and 60 min respectively while addressing the clinical trustworthiness of our model. Besides, the multimodal architectures had lower MAPE for predicting interstitial glucose compared to unimodal architectures in hypoglycaemic as well as in hyperglycaemic range. The model had limitations due to the non-availability of baseline physiological information for every patient along with the lower number of participants in the study. Therefore, as our planned future work, we aim to develop the methodologies to augment missing information based on probabilistic distribution of the dataset. Nevertheless, this model managed to predict the interstitial glucose for prediction horizon of up to 60 min with adequate prediction accuracy which can serve as a first step for generalized interstitial glucose prediction model. Besides, we also aim to conduct the studies based on impact of meal and exercises on interstitial glucose variation.

## Supplementary Information

Supplementary Information.

## Data Availability

The data that support the findings of this study are available upon reasonable request to the pilot managers from University Hospital of Larisa such as Alexandra Bargiota (abargio@med.uth.gr) and University of Thessaly such as George E Dafoulas (gdafoulas@uth.gr).

## Code availability

The code used in this study is available upon request to the corresponding author Dr. Muhammad Salman Haleem salman.haleem@warwick.ac.uk via University of Warwick license.
