# Correction: A multimodal deep learning architecture for predicting interstitial glucose for effective type 2 diabetes management

**DOI:** 10.1038/s41598-025-16371-0

**Published:** 2025-08-20

**Authors:** Muhammad Salman Haleem, Daphne Katsarou, Eleni I. Georga, George E. Dafoulas, Alexandra Bargiota, Laura Lopez-Perez, Miguel Rujas, Giuseppe Fico, Leandro Pecchia, Dimitrios Fotiadis, Gatekeeper Consortium, Claudio Caimi, Christian Tamporale, Mirko Manea, Chiara Bonferini, Eugenio Gaeta, Gloria Cea Sánchez, Ioanna Drympeta, Konstantinos Votis, Frans Folkvord, Jordi de Battle

**Affiliations:** 1 School of Engineering, University of Warwick, Coventry, CV4 7AL UK; 2 School of Electronic Engineering and Computer Science, Queen Mary University of London, London, E1 4NS UK; 3 Dept. of Materials Science and Engineering, University of Ioannina, Ioannina, Greece; 4 Faculty of Medicine, University of Thessaly, Volos, Greece; 5 Department of Endocrinology and Metabolic Diseases, University Hospital of Larisa, Larissa, Greece; 6 Universidad Politécnica de Madrid-Life Supporting Technologies Research Group, ETSIT, Madrid, Spain; 7 Università Campus Bio-Medico, Via Álvaro del Portillo, 21, 00128 Roma, Italy; 8 Hewlett-Packard Italiana, Milan, Italy; 9 Information Technologies Institute, Centre for Research and Technology Hellas, Thessaloniki, Greece; 10 PredictBy Research and Consulting, Barcelona, Spain; 11 Tilburg School of Humanities and Digital Sciences, Tilburg, The Netherlands; 12 Hospital Universitari Arnau de Vilanova and Santa Maria, Lleida, Spain; 13 Centro de Investigación Biomédica en Red de Enfermedades Respiratorias (CIBERES), Madrid, Spain

**Keywords:** Multimodal AI, Deep learning, Interstitial glucose prediction, Time series modelling

Correction to: *Scientific Reports* 10.1038/s41598-025-07272-3, published online 29 July 2025

The original version of this Article contained an error in the keywords, in which “Times series modelling” was omitted. The keywords now read: “Multimodal AI, Deep Learning, Interstitial glucose prediction, Time series modelling”.

Additionally, in the original Figure 3 and Figure 4, the information given did not align with the set variables mentioned in Table 5. The original Figure 3 and 4 and accompanying legends appear below.

**Fig. 3:**
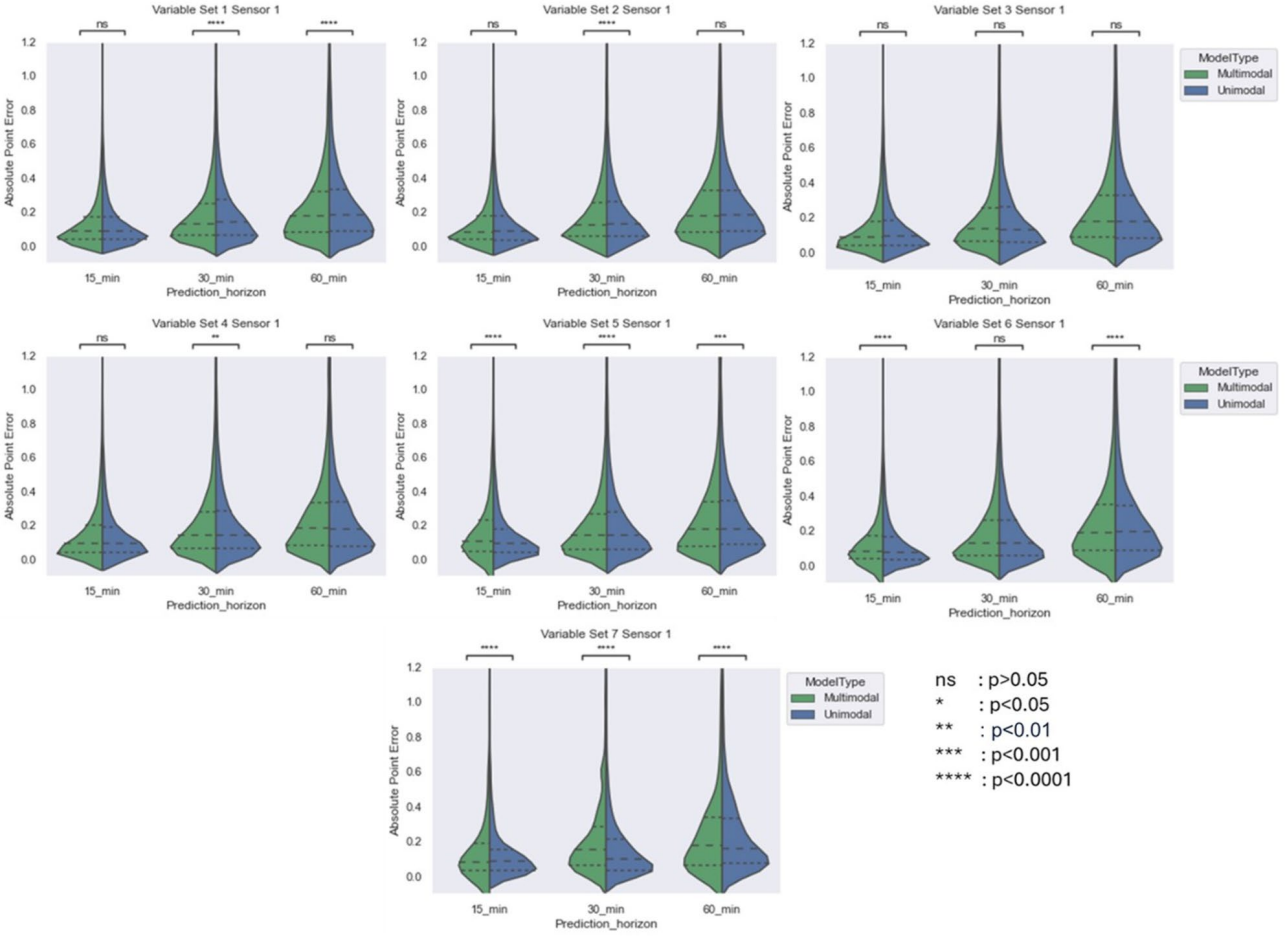
Comparing violin plot of absolute point error for multimodal and unimodal architectures developed for Menarini sensor across different variable sets at three prediction horizon. The violin plot shows the distribution of absolute point error 25%, 50% and 75% quartile via dashed line.

**Fig. 4:**
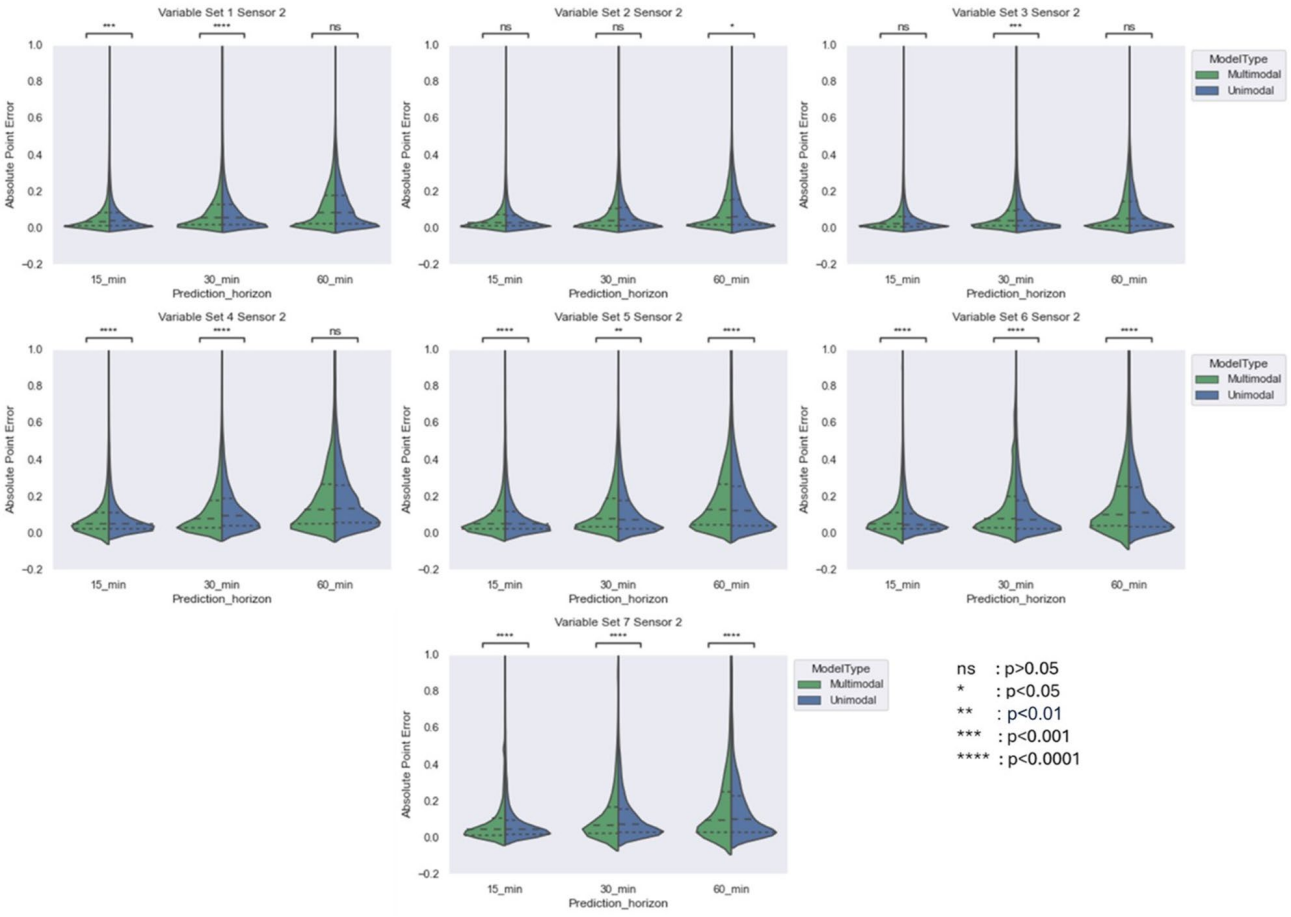
Comparing violin plot of absolute point error for multimodal and unimodal architectures developed for Abbot sensor across different variable sets at three prediction horizon. The violin plot shows the distribution of absolute point error 25%, 50% and 75% quartile via dashed line.

The original Article has been corrected.

