# Supplementary material for: A multimodal deep learning architecture for predicting interstitial glucose for effective type 2 diabetes management
Source: Sci Rep. 2025 Jul 29;15:27625. doi: 10.1038/s41598-025-07272-3 (PMC12307872; doi:10.1038/s41598-025-07272-3)
Supplement: Supplementary file 1 — Supplementary Information. [file 41598_2025_7272_MOESM1_ESM.docx]

^^[[1]](#footnote-1)^^

A Multimodal Deep Learning Architecture for Predicting Interstitial Glucose for Effective Type 2 Diabetes Management

Muhammad Salman Haleem^1,2,*^, Daphne Katsarou^3^, Eleni I. Georga^3^, George E. Dafoulas^4^, Alexandra Bargiota^5^, Laura Lopez-Perez^6^, Miguel Rujas^6^, Giuseppe Fico^6^, Leandro Pecchia^7^, Dimitrios Fotiadis^3^ and Gatekeeper Consortium

^1^School of Engineering, University of Warwick, UK

^2^School of Electronic Engineering and Computer Science, Queen Mary University of London, UK

^3^Dept. of Materials Science and Engineering, University of Ioannina, Greece

^4^Faculty of Medicine, University of Thessaly, Greece

^5^Department of Endocrinology and Metabolic Diseases, University Hospital of Larisa, Greece

^6^Universidad Politécnica de Madrid-Life Supporting Technologies Research Group, ETSIT, Madrid, Spain

^7^Università Campus Bio-Medico, Via Álvaro del Portillo, 21, 00128 Roma, Italy

*M.S.Haleem is with School of Electronic Engineering and Computer Science, Queen Mary University of London, E1 4NS, UK and School of Engineering, University of Warwick, CV4 7AL, UK. Email: [salman.haleem@warwick.ac.uk](mailto:salman.haleem@warwick.ac.uk) and [m.haleem@qmul.ac.uk](mailto:m.haleem@qmul.ac.uk)

##### Supplementary Information A.

**GATEKEEPER Consortium**

Claudio Caimi^8^, Christian Tamporale^8^, Mirko Manea^8^, Chiara Bonferini^8^, Eugenio Gaeta^6^, Gloria Cea Sánchez^6^, Ioanna Drympeta^9^, Konstantinos Votis^9^, Frans Folkvord^10,11^, Jordi de Battle^12,13^

^8^Hewlett-Packard Italiana, Milan, Italy. ^9^Information Technologies Institute, Centre for Research and Technology Hellas, Thessaloniki, Greece. ^10^PredictBy Research and Consulting, Barcelona, Spain. ^11^Tilburg School of Humanities and Digital Sciences, Tilburg, Netherlands. ^12^Hospital Universitari Arnau de Vilanova and Santa Maria, Lleida, Spain. ^13^Centro de Investigación Biomédica en Red de Enfermedades Respiratorias (CIBERES), Madrid, Spain.

##### Supplementary Information B.

This manuscript has used the TRIPOD (Transparent Reporting of a multivariable prediction model for Individual Prognosis Or Diagnosis) statement [1] to ensure a good reporting of the development of the prediction model used in this study. In particular, the TRIPOD+AI updated guidelines have been followed, as the model developed in this work is a machine learning method. The TRIPOD+AI checklist of items are represented in the overall manuscript as follows:

Supplementary Table 1. TRIPOD AI statement for Multimodal Deep Learning Architecture

| **TRIPOD+AI item** | **Manuscript section** |
| --- | --- |
| 1 (Title) & 2 (Abstract) | First page |
| 3a, 3b, 3c (Background) & 4 (Objectives) | INTRODUCTION  METHODS: Related Work |
| 5a-5b (Data) & 6a-6c (Participants) & 10 (Sample size) | METHODS: Study Protocol, Data Curation and Preprocessing |
| 7 (Data preparation) | METHODS: Data Curation and Preprocessing |
| 8a-8c (Outcome) & 15 (Model output) | METHODS: Output definition |
| 9a-9c (Predictors) | METHODS: Multimodal architecture for CGM prediction |
| 11 (Missing data) | METHODS: Data Curation and Preprocessing |
| 12a-12g (Analytical methods) &16 (Training versus evaluation) | METHODS: Model Development, Clinical Explainability of Prediction Performance |
| 18a-f (Open science items: funding, conflicts of interest, protocol, registration, data sharing, code sharing ) | Acknowledgment |
| 20a-20c (Participants) | RESULTS: Participants, Data Curation and Preprocessing, CGM prediction |
| 21 (Model development), 22 (Model specification) | RESULTS: CGM prediction |
| 23a-23b (Model performance) | RESULTS: CGM prediction, Clinical Explainability of Prediction Performance |
| 25 (Interpretation), 26 (Limitations), 27a-c (Model usability) | DISCUSSION |

##### Supplementary Information C.

This is the timeline for the study protocol, through which data has been acquired. This includes ethical approval (approved by Institutional Review Board of Larisa University Hospital), study design, data acquisition and integration into GATEKEEPER high performance big data platform


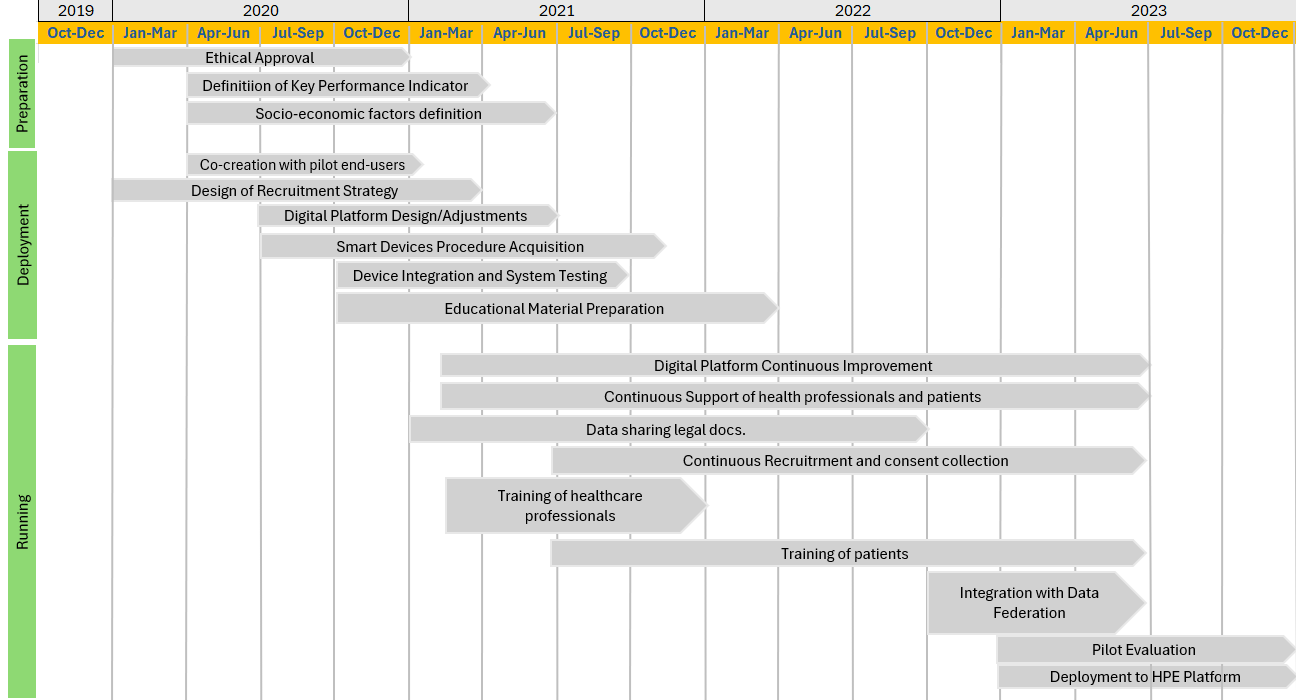


Supplementary Figure 1. Timeline for study protocol, ethical approval, data acquisition and experimentation

[1] G. S. Collins *et al.*, "TRIPOD+ AI statement: updated guidance for reporting clinical prediction models that use regression or machine learning methods," *bmj,* vol. 385, 2024.

1. [↑](#footnote-ref-1)
